# Cognitive function, body mass index and mortality in a rural elderly Chinese cohort

**DOI:** 10.1186/2049-3258-72-9

**Published:** 2014-03-26

**Authors:** Sujuan Gao, Yinlong Jin, Frederick W Unverzagt, Yibin Cheng, Liqin Su, Chenkun Wang, Feng Ma, Ann M Hake, Carla Kettler, Chen Chen, Jingyi Liu, Jianchao Bian, Ping Li, Jill R Murrell, Daniel O Clark, Hugh C Hendrie

**Affiliations:** 1 Department of Biostatistics, Indiana University School of Medicine, 410 West 10th Street, #3000, Indianapolis IN 46202-2872, Indiana; 2 Department of Psychiatry, Indiana University School of Medicine, Indianapolis, Indiana; 3 Department of Neurology, Indiana University School of Medicine, Indianapolis, Indiana; 4 Department of Pathology and Laboratory Medicine, Indiana University School of Medicine, Indianapolis, Indiana; 5 Indiana University Center for Aging Research, Indianapolis, Indiana; 6 Regenstrief Institute, Inc, Indianapolis, Indiana; 7 Eli Lilly and Company, Indianapolis, Indiana; 8 Institute for Environmental Health and Related Product Safety, Chinese Center for Disease Control and Prevention, Beijing, China; 9 Shandong Institute for Prevention and Treatment of Endemic Disease in China, Jinan, China; 10 Sichuan Provincial Center for Disease Control and Prevention in China, Chengdu, China

## Abstract

**Background:**

Previous studies have shown that poor cognition and low body mass index were associated with increased mortality. But few studies have investigated the association between cognition and mortality across the entire cognitive spectrum while adjusting for BMI. The objective of this study is to examine the associations between cognitive function, BMI and 7-year mortality in a rural elderly Chinese cohort.

**Methods:**

A prospective cohort of 2,000 Chinese age 65 and over from four rural counties in China were followed for 7-years. Cognitive function, BMI and other covariate information were obtained at baseline. Cox’s proportional hazard models were used to determine the effects of cognitive function and BMI on mortality risk.

**Results:**

Of participants enrolled, 473 (23.7%) died during follow-up. Both lower cognitive function (HR = 1.48, p = 0.0049) and lower BMI (HR = 1.6, p < 0.0001) were independently associated with increased mortality risk compared to individuals with average cognitive function and normal weight. Higher cognitive function was associated with lower mortality risk (HR = 0.69, p = 0.0312). We found no significant difference in mortality risk between overweight/obese participants and those with normal weight.

**Conclusions:**

Cognitive function and BMI were independent predictors of mortality risk. Intervention strategies for increasing cognitive function and maintaining adequate BMI may be important in reducing morality risk in the elderly population.

## Background

Increased mortality risk in the elderly population has been shown to be associated with a number of factors including older age, male gender, cardiovascular disease (CVD) and other illnesses [1]. While many previous studies have examined the association between body mass index (BMI) and mortality and between cognitive function and mortality, few have data with both cognitive and BMI measures to determine the combined effects from cognition and BMI on mortality risk. In particular, previous research has found that individuals with lower cognitive function were at increased mortality risk which was often attributed to cognitive impairment, dementia and Alzheimer’s disease [2,3]. Few studies, however, have investigated the association between cognition and mortality across the entire cognitive spectrum. Theoretically, high cognitive function can indicate larger cognitive reserve that may allow individuals with larger reserves to maintain normal functions despite of similar brain disease pathology as those with smaller reserves [4]. To the best of our knowledge, no study to date has addressed the risk of mortality in older adults with high cognitive function.

Similar to the relationship between low cognition and mortality, prior studies have also shown that low body mass index (BMI) is associated with increased mortality [5]. However, the precise relationship between BMI and mortality remains uncertain. A study in one and half million white adults found a U-shaped relationship between BMI and mortality such that individuals at both ends of the BMI scale, *i.e.* those underweight and those overweight or obese had increased mortality risk when compared to those with normal weight [6]. However, a recent meta-analysis of 97 studies reported lower mortality among overweight and moderately obese individuals relative to those with normal weight [7]. One hypothesis aimed at explaining this counterintuitive finding of lower risk associated with higher BMI in this meta-analysis and other studies [8] is that a high BMI may provide physiological reserve thus conferring protection against potential disease or illnesses [9,10].

Thus, BMI and cognition may have similar relationships to survival in the elderly population as both may indicate levels of reserve, one physiological and the other cognitive. The objectives of this study are to examine the associations between cognitive function, BMI and 7-year mortality in a rural elderly Chinese cohort.

## Methods

### Study population

Two thousand Chinese age 65 and over from four counties in China were enrolled in this study between December 2003 and May 2005. Two counties were from Sichuan province in southwestern China and two counties were from Shandong province in eastern China. For each village included in the study, the Chinese investigators and a team of interviewers who were employees of provincial and county Center for Disease Control traveled to the area, established a temporary headquarter and conducted a complete census of residents over age 65 in the area. They enrolled eligible residents by going door-to-door, obtaining informed consent before conducting interviews and collecting biological samples. There were no refusals. Since there were outreach activities by employees from the county offices of the Chinese Center for Disease Control to prepare each community for the surveys, no refusal was not surprising and it was not unusual for studies conducted in rural China, as reported in our pilot study [11] and a large dementia prevalence study [12]. However, a few subjects with hearing problems were not enrolled. The study was approved by Indiana University Institutional Review Board and the Institute for Environmental Health and Related Safety, Chinese Center for Disease Control and Prevention. Details of the study were described previously [13].

### Endpoints

Cognitive assessment was conducted at baseline enrollment. Two follow-up evaluations were conducted at 2.5 and 7 years after the baseline assessment. For participants who had died, interviews were conducted with a close relative to obtain date of death. Nine participants had died but their relatives were not able to provide death date; thus we used the median time point between their last evaluation time and date of the follow-up evaluation as an estimate for date of death. For surviving participants, censoring time was defined as the last time they were evaluated.

### Cognitive assessment

Cognitive assessment was conducted in face-to-face interviews using six cognitive tests: the Community Screening Instrument for Dementia (CSID), CERAD 10-word list learning, word-list recall [14], IU Story Recall, Animal Fluency test [15], and IU Token test. The CSID was developed as a screening tool for dementia in populations with various cultural backgrounds and literacy levels. Details of the instrument have been published elsewhere [16]. In this analysis, a composite cognitive z-score was derived by using the average of standardized scores of the six cognitive tests.

### BMI measures

Participants’ height in meters and weight in kilograms were measured during the baseline interview. Body Mass Index (BMI) was derived weight in kilograms divided by height in meters squared.

### Collection of other risk factors

Other risk factors collected during the baseline interview include age, gender, whether the participant attended school and years of schooling, marital status (married, widowed or other), household composition (living with spouse, living with children, living with spouse and children, or other), alcohol consumption and smoking history (yes or no), history of cancer, Parkinson’s disease, diabetes, hypertension, stroke, heart attack, head injury and bone fracture. Participants’ blood pressure (2 times) was also measured during the interview. The average of the two blood pressure measures were used in our analyses. Blood spots on filter paper were collected from all study participants at the end of the interview. Apolipoprotein E (*APOE*) genotype was determined by eluting DNA from a dried blood spot [17] followed by *Hhal digestAPOEion* of amplified products [18].

### Statistical analysis

In order to capture potential nonlinear association between baseline cognitive function and mortality, we divided the study population into quintiles according to baseline cognitive *z*-score levels. BMI was modeled using the World Health Organization criteria into four groups: underweight (BMI < 18.5), normal weight (18.5 ≤ BMI < 25), overweight (25 ≤ BMI < 30), and obese (BMI ≥ 30) [19]. The following variables were considered to be potential confounders possibly related to mortality: age at baseline, gender, education (whether the participant attended school), marital status, household composition, alcohol consumption and smoking history, systolic and diastolic blood pressure measures, *APOE* genotype (ϵ4 carriers vs. non-carriers) and history of comorbidities, such as cancer, Parkinson’s disease, diabetes, hypertension, stroke, heart attack, head injury and fracture.

Analysis of variance models (ANOVA) were used to compare differences in continuous variables, and chi-square tests were used to compare differences in categorical variables across the five quintile groups defined by cognitive *z*-scores. Kaplan-Meier estimator was used to estimate survival probabilities for the cohort stratified by cognitive quintile and BMI groups, respectively. Univariate Cox’s proportional hazard model was used to examine the association between each covariate and mortality risk. Multivariate Cox’s proportional hazard model was used to explore the association between cognitive scores, BMI and mortality adjusting for significant covariates identified in univariate models.

## Results

Of the 2000 participants enrolled at baseline, 144 died prior to the 2.5 year follow-up evaluation and 329 participants died between the 2.5 year and the 7 year follow-up evaluations. In Table 1, we present baseline characteristics of study participants by cognitive quintile groups. Age, gender, education, marital status, household composition, smoking, consuming alcohol, BMI, history of hypertension and heart attack were significantly different among cognitive quintile groups. In particular, participants in the lowest cognitive quintile group were more likely to be underweight and those with the highest cognitive scores were more likely to be overweight or obese.

**Table 1:** Participants’ baseline characteristics by quintile groups of composite cognitive scores, Selenium and Cognitive Decline Study, People’s Republic of China, December 2003 – April 2012

	**Cognitive quintile groups**					
	**Quintile 1**	**Quintile 2**	**Quintile 3**	**Quintile 4**	**Quintile 5**	
	**(n = 400)**	**(n = 400)**	**(n = 400)**	**(n = 400)**	**(n = 400)**	**p value**
Mean age, years (SD)	75.3(6.1)	72.8(5.6)	71.6(5.3)	70.5(4.5)	69.5(4.3)	**<.0001**
Female (%)	297(74.3)	240(60.0)	226(56.5)	184(46.0)	132(33.0)	**<.0001**
Ever attended school (%)	52(13.0)	109(27.3)	145(36.3)	177(44.3)	270(67.5)	**<.0001**
Marital status (%)						**<.0001**
Married	198(49.5)	231(57.8)	252(63.0)	276(69.0)	294(73.5)	
Widowed	201(50.3)	165(41.3)	142(35.5)	118(29.5)	100(25.0)	
Other	1(0.3)	4(1.0)	6(1.5)	6(1.5)	6(1.5)	
Household composition (%)						**<.0001**
Live with spouse	152(38.0)	187(46.8)	194(48.5)	232(58.0)	248(62.0)	
Live with children	158(39.5)	114(28.5)	120(30.0)	87(21.8)	66(16.5)	
Live with spouse and children	22(5.5)	24(6.0)	21(5.3)	26(6.5)	28(7.0)	
Other	68(17.0)	75(18.8)	65(16.3)	55(13.8)	58(14.5)	
Smoking (%)	128(32.0)	155(38.8)	195(48.8)	209(52.3)	241(60.3)	**<.0001**
Consume alcohol (%)	133(33.3)	157(39.3)	183(45.8)	180(45.0)	220(55.0)	**<.0001**
Body mass index, kg/m^2^	20.9(3.2)	21.5(3.3)	21.9(3.4)	22.5(3.5)	22.9(3.8)	**<.0001**
BMI groups:						
Underweight	85(21.3)	59(14.8)	53(13.3)	36(9.0)	39(9.8)	**<.0001**
Normal weight	274(68.5)	291(72.8)	277(69.3)	286(71.5)	257(64.3)	
Overweight	37(9.3)	43(10.8)	60(15.0)	66(16.5)	88(22.0)	
Obese	4(1.0)	7(1.8)	10(2.5)	12(3.0)	16(4.0)	
Systolic blood pressure, mmHg (SD)	145.2(25.3)	144.0(23.4)	145.9(25.1)	147.1(24.7)	146.2(26.0)	0.4693
Diastolic blood pressure, mmHg (SD)	83.1(12.8)	83.2(12.3)	83.7(13.1)	84.2(12.3)	84.0(12.7)	0.6546
History of (%)						
Cancer	0(0.0)	3(0.8)	3(0.8)	3(0.8)	4(1.0)	0.4686
Parkinson’s disease	5(1.3)	1(0.3)	4(1.0)	6(1.5)	2(0.5)	0.3062
Diabetes	10(2.5)	10(2.5)	9(2.3)	9(2.3)	14(3.5)	0.7911
Hypertension	228(57.0)	222(55.5)	235(58.8)	254(63.5)	231(57.8)	0.1936
Stroke	16(4.0)	7(1.8)	18(4.5)	10(2.5)	11(2.8)	0.1492
Heart attack	12(3.0)	8(2.0)	14(3.5)	11(2.8)	24(6.0)	**0.0247**
Head injury	28(7.0)	17(4.3)	29(7.3)	21(5.3)	23(5.8)	0.3465
Fracture	7(1.8)	9(2.3)	14(3.5)	9(2.3)	12(3.0)	0.5476
APOE genotype, ϵ4 carriers (%)	84(21.0)	66(16.5)	60(15.0)	63(15.8)	60(15.0)	0.1226

p-values in bold indicate significance at the 0.05 level.

**Figure 1:**
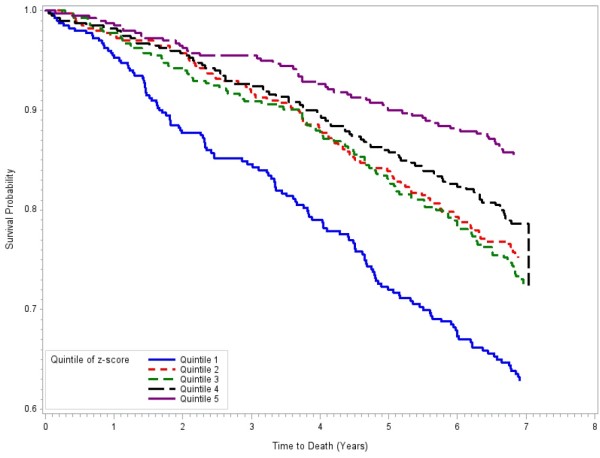
Kaplan-Meier Survival Curves by Cognitive Quintile Groups, Selenium and Cognitive Decline Study, People’s Republic of China, December 2003 – April 2012.

**Figure 2:**
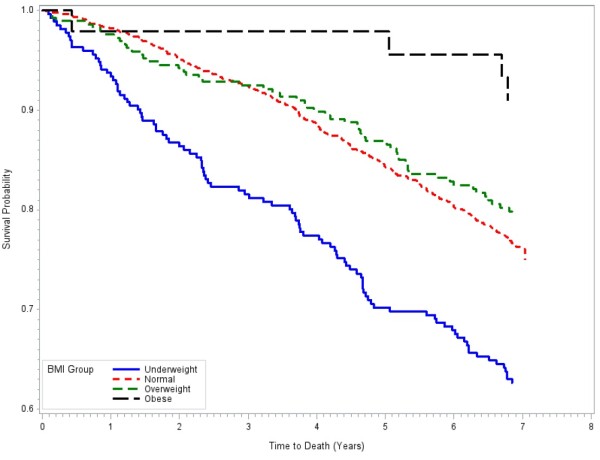
Kaplan-Meier Survival Curves by Body Mass Index Groups, Selenium and Cognitive Decline Study, People’s Republic of China, December 2003 – April 2012.

Kaplan-Meier survival estimates by cognitive quintile groups were presented in Figure 1. There were significant differences in survival probabilities among the five cognitive quintile groups (p < 0.0001). Participants with the lowest cognitive scores had the lowest survival probability and those with the highest cognitive scores had the best survival probability while quintile groups 2, 3 and 4 showed similar survival probabilities. Kaplan-Meier survival estimates by BMI groups were presented in Figure 2. Participants in the underweight group had the lowest survival probability while the obese group seems to have higher survival, although there were fewer deaths in that group making the survival curve for that group unstable. The overweight participants showed similar survival probability to those with normal weight.

In univariate Cox’s models, we found that increasing age, male gender, being widowed, living with children, smokers, drinkers, lower BMI, history of Parkinson’s disease, diabetes, stroke, or lower cognitive function were each significantly associated with lower survival probability. *APOE* ϵ4 carrier status was not associated with mortality risk in univariate Cox’s model (HR = 1.01, p = 0.9296). In Table 2 we present the multivariate Cox’s model with both cognitive quintile groups and BMI groups. Participants with the lowest cognitive scores, *i.e.* those in the 1st quintile group, had 47.5% increase in mortality compared to those with average cognitive score (3rd quintile group). Participants with the highest cognitive scores, on the other hand, had a 31.4% reduction in hazard rate of death. Participants who were underweight at baseline had increased mortality risk (HR = 1.6, p < 0.0001) compared to those with normal weight. Individuals in the overweight group or the obese group were not significantly different in hazard rate from those in the normal weight group.

**Table 2:** Results of Multivariate Cox’s Proportional Hazard Model, Selenium and Cognitive Decline Study, People’s Republic of China, December 2003 – April 2012

**Variables**	**Entire cohort (n = 2000)**		Sub-sample (n = 1948)^*^	
	**Hazard ratio**	**95% CI**	**Hazard ratio**	**95% CI**
Age at Baseline	1.096	(1.080-1.113)	1.095	(1.078-1.113)
Female	0.579	(0.478-0.701)	0.542	(0.442-0.664)
BMI:				
Underweight	1.639	(1.301-2.064)	1.504	(1.170-1.933)
Overweight	1.092	(0.818-1.457)	1.047	(0.770-1.424)
Obese	0.392	(0.146-1.055)	0.314	(0.100-0.984)
Normal weight	1.000	Reference	1.000	Reference
Diabetes	2.235	(1.390-3.595)	2.303	(1.391-3.814)
Stroke	1.777	(1.142-2.765)	1.954	(1.241-3.077)
Cognitive quintiles				
1st Quintile	1.475	(1.130-1.926)	1.165	(0.878-1.545)
2nd Quintile	1.241	(0.936-1.645)	0.791	(0.588-1.064)
3rd Quintile	1.000	Reference	1.000	Reference
4th Quintile	1.074	(0.803-1.439)	0.851	(0.628-1.154)
5th Quintile	0.686	(0.493-0.955)	0.537	(0.380-0.759)

^*^Excluding individuals who died within one year of baseline evaluation.

In order to determine whether the increased mortality for the underweight participants or those with poor cognition is due to severe illness, we repeated the multivariate Cox’s models excluding individuals who died within one year of their baseline evaluation. The effect of underweight was attenuated, but remained significant (HR = 1.465, p < 0.0028) compared to those with normal weight. However, there is no longer an increased risk from the poor cognition group. Given results obtained in the entire sample where the poor cognition group had increased mortality risk, it seems to indicate that those in the lowest cognitive quintile were more likely to die within one year of the baseline. The protective effect in the highest cognitive score quintile group remained significant as seen in the entire cohort. There is no significant interaction between cognitive function and BMI on mortality in the entire sample (p = 0.3276) or the sub-sample (n = 0.5874).

## Discussion

In this rural elderly Chinese cohort followed for seven years, we found that both lower cognitive function and lower BMI are independently associated with increased mortality risk compared to individuals with average cognitive function and normal weight. In addition, we found that higher cognitive function is associated with lower mortality risk. At the same time, we found no significant difference in mortality risk between overweight/obese participants and those with normal weight.

Previous studies have reported increased mortality risk of individuals with poor cognitive function and have attributed the increased risk to cognitive impairment and dementia [3,20-23]. When we excluded participants who died within the first year of baseline, poor cognition was no longer significantly associated with increased mortality, suggesting that terminal illnesses may be a cause for poor cognition [2]. However, few studies have separately examined mortality risk in individuals with higher than average cognitive scores. Our result of lower mortality risk in individuals from the top cognitive quintile group compared to those with average cognitive scores suggests a potential protective mechanism of high cognitive function in the absence of disease pathology. This protective effect could result from more years of formal education, a more intellectually stimulating job, life-long pursuit of leisure activities with cognitive benefit, or healthier lifestyles [24]. In our study, individuals with the highest cognitive function also had the highest proportion of schooling, although education itself was not an independent predictor of mortality in the final multivariate model. It is also interesting to observe that the highest cognitive functioning individuals in our cohort had similar levels of medical comorbidities as participants in the other cognitive groups suggesting that the lower mortality risk in the high cognition group is unlikely due to better physical health in this group.

In terms of BMI, our results support previous findings of increased mortality risk for underweight participants as previously reported in a large Chinese study that also included younger study subjects (mean age 52 years) [25], in white adults [6], and in non-Hispanic black adults [26]. The increased mortality risk in underweight individuals was thought to be due to increased comorbidity burdens in these individuals. However, our final multivariate models included diabetes, stroke and cognitive function and still found significantly increased mortality hazard for the underweight participants, hence these comorbid conditions are unlikely to account for the increased mortality in this group. The underweight individuals were also suspected to be suffering from terminal diseases that resulted in weight loss. However, when we tested this hypothesis by excluding individuals who died within one year of baseline evaluation, we still observed an increased mortality risk in the underweight individuals. In fact, the survival plot in Figure 2 seems to suggest a steady rate of death in the underweight group across the seven years of follow-up.

While our analyses lend support to a protective effect of cognitive reserve, our results are inconclusive on the effect of a physiologic reserve. Unlike previous studies [6,25,26], we did not find an increased mortality risk for those overweight or obese. We also did not find lower mortality among those overweight and obese participants as reported in other studies [7]. Since this cohort has few participants with BMI in the obese range, we anticipated insufficient power to examine the risk associated with obesity. Nevertheless, the survival plot in Figure 2 and the hazard ratio estimate for the overweight group suggest that there are little differences in mortality risk between the overweight and normal weight participants. Thus for physiologic reserves defined by BMI, our results support the maintenance of normal weight in the elderly, but offer no evidence of additional protection from BMI higher than normal weight on mortality risk.

Other predictors that have been consistently shown to be risk factors for mortality include older age, male gender, history of diabetes and cardiovascular diseases [27]. Interestingly, in our cohort education was not associated with mortality after adjusting for cognitive function, although the majority of our cohort has little or no education (62%). The low variation in education levels among our participants may explain the lack of association between education and mortality. Alternatively, the effect of education may be attenuated in the presence of cognitive function in the survival models.

Results on the relationship between *APOE* ϵ4 genotype and mortality risk have not been consistent as some studies found increased mortality risk in *APOE*ϵ*4* carriers [28,29] while others reported no relationship [30,31]. The discrepancy in results was sometimes attributed to whether cognitive status was adjusted in the models or to low ϵ*4* allele frequencies in the population studied [27]. In our cohort, *APOE*ϵ*4* was not a significant predictor of mortality even without adjusting for cognitive function. However, this cohort does have low *APOE*ϵ*4* frequency (8.8%) [13] which may account for the non-significant relationship between *APOE*ϵ*4* and mortality.

Our study has a number of strengths. The first is our large cohort size with relatively long follow-up. A comprehensive set of cognitive measures were used in our study ensuring robust results in grouping participants according to their composite cognitive scores. The cohort is also unique as few studies with cognitive and body composition measures have been conducted in rural elderly Chinese.

This study also has important limitations. Our analysis focused on all causes mortality as many of our participants died at home and accurate information on causes of death was not available. It is also possible that other predictors of mortality, especially socio-economic information other than education, were not included in our models.

## Conclusions

In summary, in this rural elderly Chinese cohort followed over seven years, baseline cognitive function and BMI were independent predictors of mortality risk. We found that underweight participants had increased mortality risk compared to participants with normal weights. In addition, we found that individuals with high cognition had decreased mortality risk. Our results suggest that maintaining adequate BMI may be a more important priority in reducing mortality risk in the elderly population than implementing weight loss programs. Our results also indicate that increasing cognitive reserves through education and improved literacy to maintaining high cognitive function in late life is important in reducing mortality risk.

## Competing interests

The authors declare that they have no competing interests.

## Authors’ contributions

SG, YJ, FWU, HCH participated in the design of the study. YJ, YC, LS, FM, CC, JL, AMH, JRM, JB and PL supervised the data collection. SG and CW performed the statistical analysis and drafted the manuscript. All authors read and approved the final manuscript.
